# Urinary Catecholamines Predict Relapse During Complete Remission in High-Risk Neuroblastoma

**DOI:** 10.1200/PO-24-00491

**Published:** 2025-02-21

**Authors:** Yvette A.H. Matser, Atia Samim, Marta Fiocco, Marieke van de Mheen, Maria van der Ham, Monique G.M. de Sain-van der Velden, Nanda M. Verhoeven-Duif, Martine van Grotel, Kathelijne C.J.M. Kraal, Miranda P. Dierselhuis, Natasha K.A. van Eijkelenburg, Karin P.S. Langenberg, Max M. van Noesel, André B.P. van Kuilenburg, Godelieve A.M. Tytgat

**Affiliations:** ^1^ Princess Máxima Center for Pediatric Oncology, Utrecht, the Netherlands; ^2^ Division of Imaging and Oncology, University Medical Center, Utrecht, the Netherlands; ^3^ Department of Biomedical Data Science, Section Medical Statistics, Leiden University Medical Centre, Leiden, the Netherlands; ^4^ Mathematical Institute, Leiden University, Leiden, the Netherlands; ^5^ Department of Genetics, Section Metabolic Diagnostics, Wilhelmina Children's Hospital, Utrecht, the Netherlands; ^6^ Laboratory Genetic Metabolic Diseases, Amsterdam UMC, Free University Amsterdam, Amsterdam, the Netherlands; ^7^ Cancer Center Amsterdam, Imaging and Biomarkers, Amsterdam, the Netherlands; ^8^ Department of Genetics, Utrecht University Medical Center, Utrecht University, Utrecht, the Netherlands

## Abstract

**PURPOSE:**

Urinary catecholamine metabolites are well-known biomarkers for the diagnosis (Dx) of neuroblastoma, but their clinical significance in determining therapy response during treatment is not well established. Therefore, catecholamines are not included in criteria for assessing response and complete remission (CR). This study investigated the use of urinary catecholamines in response monitoring and predicting survival outcomes.

**METHODS:**

From 2005 to 2021, a panel of eight urinary catecholamines were measured in patients with high-risk neuroblastoma at Dx and at standard evaluation moments during treatment. At the same time points, response and CR were assessed according to the revised International Neuroblastoma Response Criteria.

**RESULTS:**

The total cohort consists of 153 high-risk patients, and at least one of the eight metabolites was elevated (ie, catecholamine status positive) in 141 of 146 (97%), 104 of 128 (81%), and 39 of 69 (57%) patients at Dx, postinduction, and at CR, respectively. Primary tumor resection significantly reduced catecholamine levels (*P* < .01). A positive catecholamine status at Dx, during treatment, and at the end of treatment was not significantly associated with event-free survival (EFS) or overall survival (OS). However, in patients who achieved CR, those with a positive catecholamine status had poor EFS (38% *v* 80%, respectively; *P* < .01) and OS (52% *v* 86%, respectively; *P* = .01) compared with those with a negative catecholamine status. Notably, 3-methoxytyramine levels at CR seem to be a prognostic marker for poor OS (hazard ratio, 7.5 [95% CI, 2.0 to 28.6]).

**CONCLUSION:**

Catecholamine measurements contribute to the assessment of CR and identifies patients with high-risk neuroblastoma with an increased risk of relapse and death.

## INTRODUCTION

Neuroblastoma is a solid pediatric malignancy that arises from the adrenal medulla or the sympathetic side chain.^1^ Despite intensive treatments, 50%-60% of patients with high-risk neuroblastoma experience relapse and 90% of these patients will die from the disease.^2,3^ Several factors that are predictive of poor outcome have been identified in high-risk neuroblastoma, such as segmental chromosomal aberrations, age at diagnosis (Dx), meta-[^123^I]iodobenzylguanidine (MIBG) scoring, and minimal residual disease detection in blood or bone marrow samples.^4-8^ However, there is a lack of biomarkers for prediction of poor outcome that are easy to measure and noninvasive to collect over time.

**CONTEXT**

**Key Objective**
Half of all patients with high-risk neuroblastoma who achieve complete remission (CR) will experience relapse of disease. Detection of catecholamine metabolites in urine is a sensitive standard-of-care approach to diagnose patients with neuroblastoma. However, catecholamines are not included in standardized response evaluations or CR definitions. Catecholamine metabolites offer a noninvasive method to monitor tumor response and predict patient survival.
**Knowledge Generated**
An eight-marker urinary catecholamine panel was measured at standard treatment evaluation moments in patients with high-risk neuroblastoma. Our data showed that elevated catecholamine levels at CR predict increased risk of relapse and mortality, indicating that catecholamine measurements could enhance the current criteria for CR. Elevated levels of individual catecholamines were also associated with poor outcome at several standard evaluation moments during treatment.
**Relevance**
These results support further investigations of using catecholamine metabolites to improve response monitoring in patients with high-risk neuroblastoma and CR assessments.

One of the characteristics of neuroblastoma tumors is the production of catecholamine metabolites, such as homovanillic acid (HVA) and vanillylmandelic acid (VMA).^9,10^ Despite the high excretion levels and world-wide clinical testing during treatment, they are not incorporated into the revised International Neuroblastoma Response Criteria (INRC).^11^ While HVA and VMA were included in the previous version of the INRC,^12^ they were later excluded because of a lack of standardization in measurement methods and concerns of dietary influences on metabolite excretion.^11^ To address these concerns, we demonstrated the feasibility and added clinical value of testing a panel of eight markers.^13^ Next, we showed in a Society of Paediatric Oncology European Neuroblastoma Network (SIOPEN) diagnostic study that the sensitivity of HVA and VMA for disease detection was 89% and increased to 95% when extending the panel with six additional catecholamine metabolites in a single urine portion without dietary restrictions.^14^ Furthermore, in international patient cohorts, elevated levels of the metabolite 3-methoxytyramine (3MT) at Dx were shown to strongly associate with poor outcome, even in high-risk patients.^15,16^ There are limited data on the role of catecholamine measurements during neuroblastoma treatment. Previously, increased HVA and VMA levels were not associated with poor outcome in patients with stage IV neuroblastoma after four cycles of induction chemotherapy.^17^ However, whether the extended panel of eight catecholamine metabolites is useful for detecting tumor activity and relates to poor outcome during treatment has not been investigated.

In this study, the extended eight-marker catecholamine panel was determined in patients with high-risk neuroblastoma at decision making moments during treatment. At these moments, we analyzed if the catecholamine panel was predictive of poor survival outcome. In addition, we assessed when patients achieved complete remission (CR) during treatment and evaluated if the catecholamine panel proved to be useful for detection of tumor activity, thus adding value to the current criteria for CR.

## METHODS

### Patients

Patients with high-risk neuroblastoma diagnosed at Emma Children's Hospital, Sophia Children's Hospital, or the Princess Máxima Center for Pediatric Oncology in the Netherlands between January 2005 and March 2021 were eligible. All patients were treated according to the DCOG-NBL2009 high-risk protocol. Patients who were initially diagnosed as low-risk but later upstaged to high-risk were included in the study at the time of upstaging. Informed consent was obtained from all participants or guardians. Approval for this retrospective study was granted by the local ethics committees (W16_093#16.112 and PMCLAB2019.075).

### Urine Collection and Catecholamine Measurements

**FIG A1.**
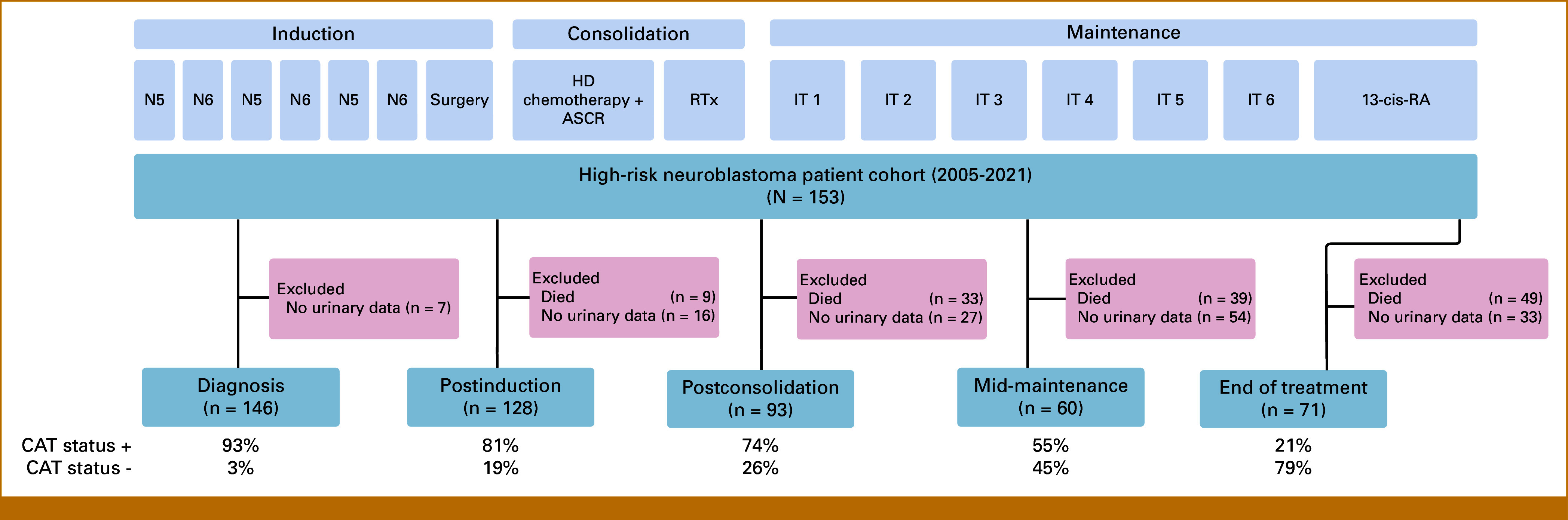
Flow diagram and neuroblastoma treatment periods. ASCR, autologous stem cell rescue; CAT, catecholamine; cis-RA, cis-retinoic acid; HD, high-dose; IT, mid-maintenance; RTx, radiotherapy.

Urine samples were routinely collected as standard-of-care response evaluation at the following time points: at Dx, end of induction (ie, postinduction [PI]), postconsolidation (PC), during maintenance (ie, mid-maintenance [IT]), and at the end of treatment (ET; Appendix Fig A1). To evaluate the additive value of catecholamine detection to establish CR, we also determined whether a patient was in CR at these evaluation time points: PI, PC, at IT, and at the ET. Response to therapy and CR were defined according to the revised INRC, which incorporates imaging and bone marrow assessments.^11^ Spot urine (ie, a random single urine collection) or 24-hour urine specimens were collected. Catecholamine metabolites were analyzed using high-performance liquid chromatography with fluorescence detection or ultra-performance liquid chromatography (UPLC)-tandem mass spectrometry (MS; UPLC-MS/MS), as previously described.^14^ Eight urinary catecholamine metabolites were measured, namely, HVA, VMA, dopamine (DA), 3MT, norepinephrine (NE), normetanephrine (NMN), epinephrine (E), and metanephrine (MN). Fold changes of catecholamine metabolite measurements were calculated by dividing the catecholamine metabolite excretion (per unit creatinine) by the 95th upper age-related reference limit of the metabolite.^13,14^ A metabolite with fold change >1 is considered elevated.^18^ If any of the eight metabolites in the panel exceeded the upper reference limit, the catecholamine panel was considered positive (ie, catecholamine status positive).

### Statistical Analysis

A competing risk model was used to estimate the cumulative incidence function of relapse from Dx, PI, PC, during maintenance, and at the ET with death as the competing event.^19^ Gray's test was used to assess the difference between cumulative incidence.^20^ Event-free survival (EFS) and overall survival (OS) were estimated from the above-described five clinical starting points by using Kaplan-Meier methodology. Moreover, to evaluate the prognostic impact of catecholamine metabolites in patients in CR, EFS (all events were relapses after CR was achieved) and OS were assessed from the point of CR. To assess the time from CR to an event, the date of CR was used as the landmark point. The log-rank test was used to assess the difference in survival outcomes. The median follow-up time was estimated using reverse Kaplan-Meier method.^21^ To assess the effect of prognostic factors on survival outcomes, a Cox proportional hazards regression model was estimated. A paired t-test was performed to evaluate the differences in individual log-transformed catecholamine metabolite levels between pre- and postsurgery. To compare the proportion of patients with a positive catecholamine metabolite before and after surgical resection of the primary tumor, a McNemar test was used. Statistical significance was defined as a *P* value of <.05. Statistical analyses were performed using GraphPad Prism (version 9.3.0). All analyses concerning survival and competing risks were performed in the R software environment by using the survival and cmprsk library.^22^

## RESULTS

### Patients and Urine Samples

This study included 153 patients with high-risk neuroblastoma with a median age at Dx of 3.3 years. Urinary catecholamine data were available for 146 of 153 patients at Dx, 128 of 144 PI, 93 of 120 PC, 60 of 114 at IT, and 71 of 104 at the ET (Appendix Fig A1). The median follow-up time from Dx was 59 months (range, 1.4-216.4). In total, 76 patients died because of progressive or relapsed disease (n = 67), treatment-related toxicity (n = 8), or secondary malignancy (n = 2; Table 1). Among the 153 high-risk patients, 120 achieved CR according to the INRC criteria, which included nuclear imaging and bone marrow assessment. Urinary data were available for 69 patients at CR (median interval between CR and urine sample sampling: 12 days; range, 1-48; Appendix Table A1).

**TABLE 1.** Clinical Characteristics

Characteristic	N = 153, Value (%)
Age at Dx, years	
<18 months	23 (15)
>18 months	130 (85)
Sex	
Male	88 (58)
Female	65 (42)
INRGSS stage	
L1	1 (1)
L2	7 (5)
M	145 (94)
Risk group	
High-risk at initial Dx	139 (91)
High-risk after upstaging	14 (9)
Reached end of treatment	
Yes, including six courses of immunotherapy	81 (53)
Yes, no immunotherapy	21 (14)
No	51 (33)
Achieved complete remission	
Yes	120 (78)
No	33 (22)
Molecular aberrations at Dx^a^	
*MYCN* amplification	54/152 (36)
Loss 1p	66/149 (44)
Gain 17q	109/123 (89)
Loss 11q	48/128 (38)
Events	
No event	59 (39)
Progression	24 (16)
Relapse	60 (39)
Death because of treatment-related toxicity	8 (5)
Secondary malignancy	2 (1)
Deceased	
Yes	76 (50)
No	77 (50)

Abbreviations: Dx, diagnosis; INRGSS, International Neuroblastoma Risk Group Staging System.

^a^ Total number of included patients is lower because of missing data regarding the molecular aberrations.

### Catecholamine Metabolites During Treatment

**FIG 1.**
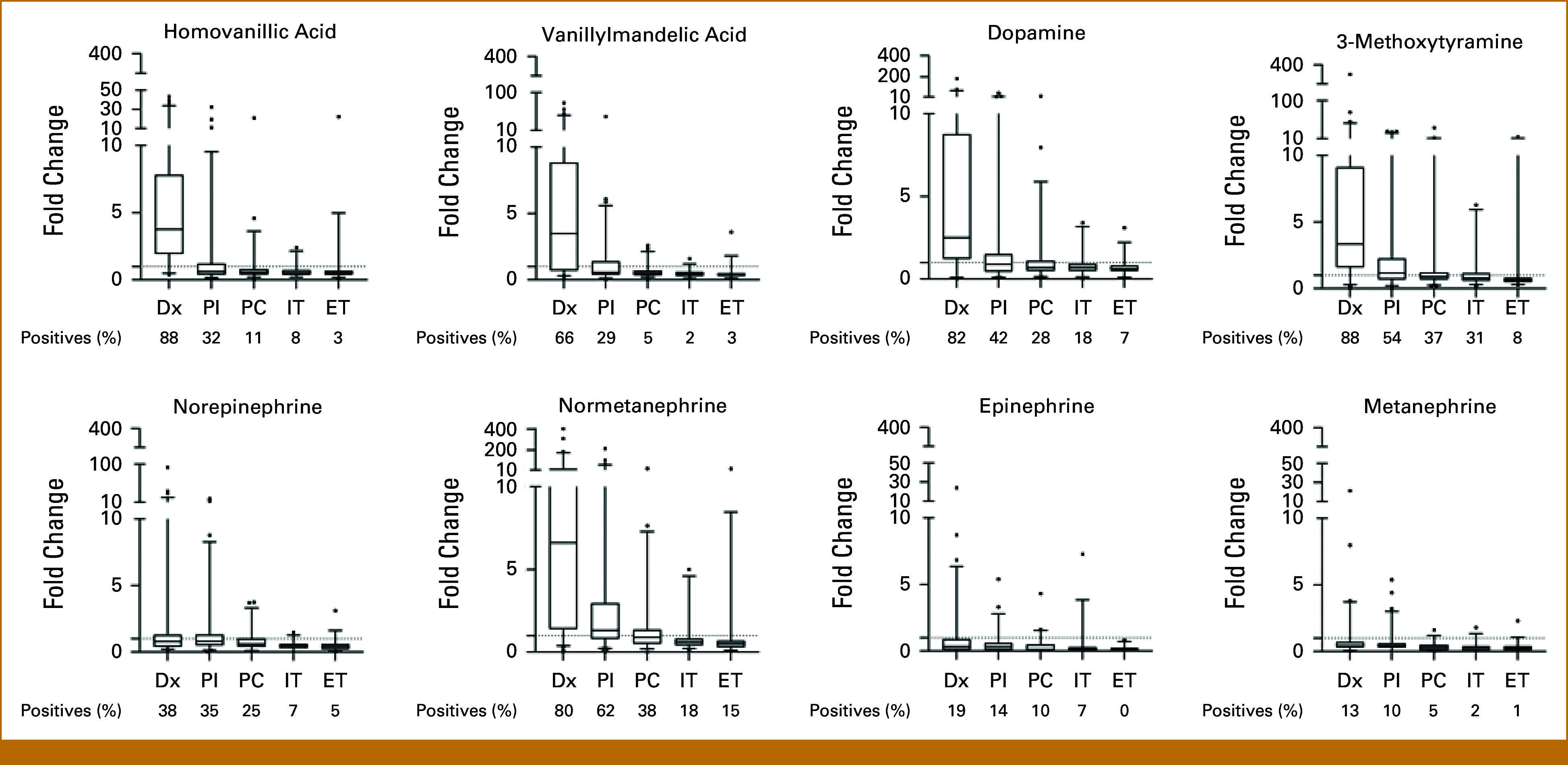
Urinary catecholamine metabolite levels at Dx and during and after therapy. For each urinary catecholamine metabolite, fold change (*y*-axis) is given for each of the five clinical time points. A fold change value >1 is considered elevated, indicated by the dotted line. Boxplots represent the median and interquartile ranges. Values above the 97.5th percentile are shown as single dots. The percentage of positives below the *x*-axis indicate the percentage of patients with positive catecholamine measurements for each time point. Dx, diagnosis; ET, end of treatment; IT, mid-maintenance; PC, postconsolidation; PI, postinduction.

**FIG 2.**
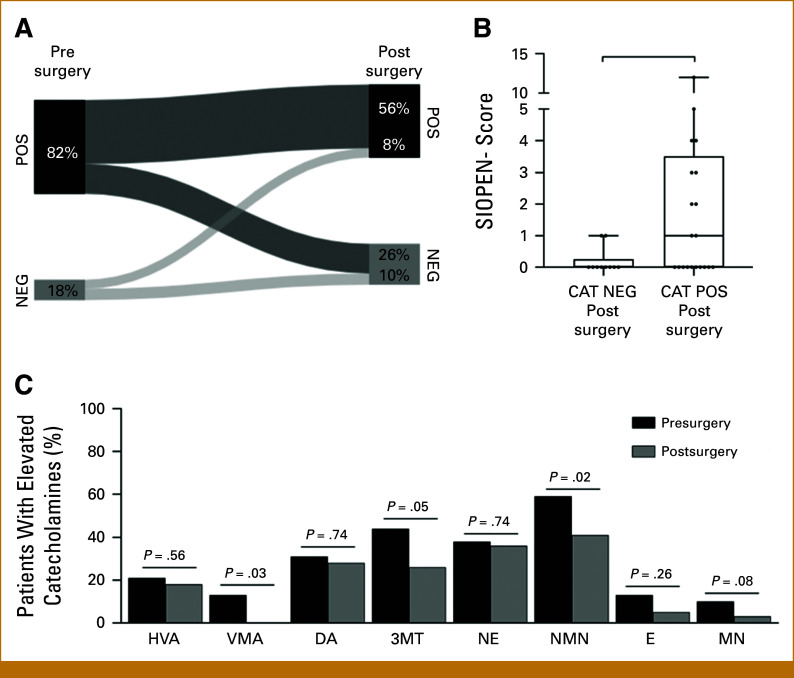
Urinary catecholamine levels before and after surgery. (A) Paired analysis of percentage of patients with POS or NEG catecholamine status and the shift between POS and NEG between pre- and postsurgery. (B) Paired postsurgery MIBG SIOPEN scores for patients with postsurgery NEG and POS catecholamine status. Boxplot with median and interquartile ranges is depicted. (C) Percentage of patients with a positive catecholamine status for each individual metabolite before and after surgery. 3MT, 3-methoxytyramine; CAT, catecholamine; DA, dopamine; E, epinephrine; HVA, homovanillic acid; MIBG, meta-[123I]iodobenzylguanidine; MN, metanephrine; NE, norepinephrine; NEG, negative; NMN, normetanephrine; POS, positive; SIOPEN, Society of Paediatric Oncology European Neuroblastoma Network; VMA, vanillylmandelic acid.

**FIG A2.**
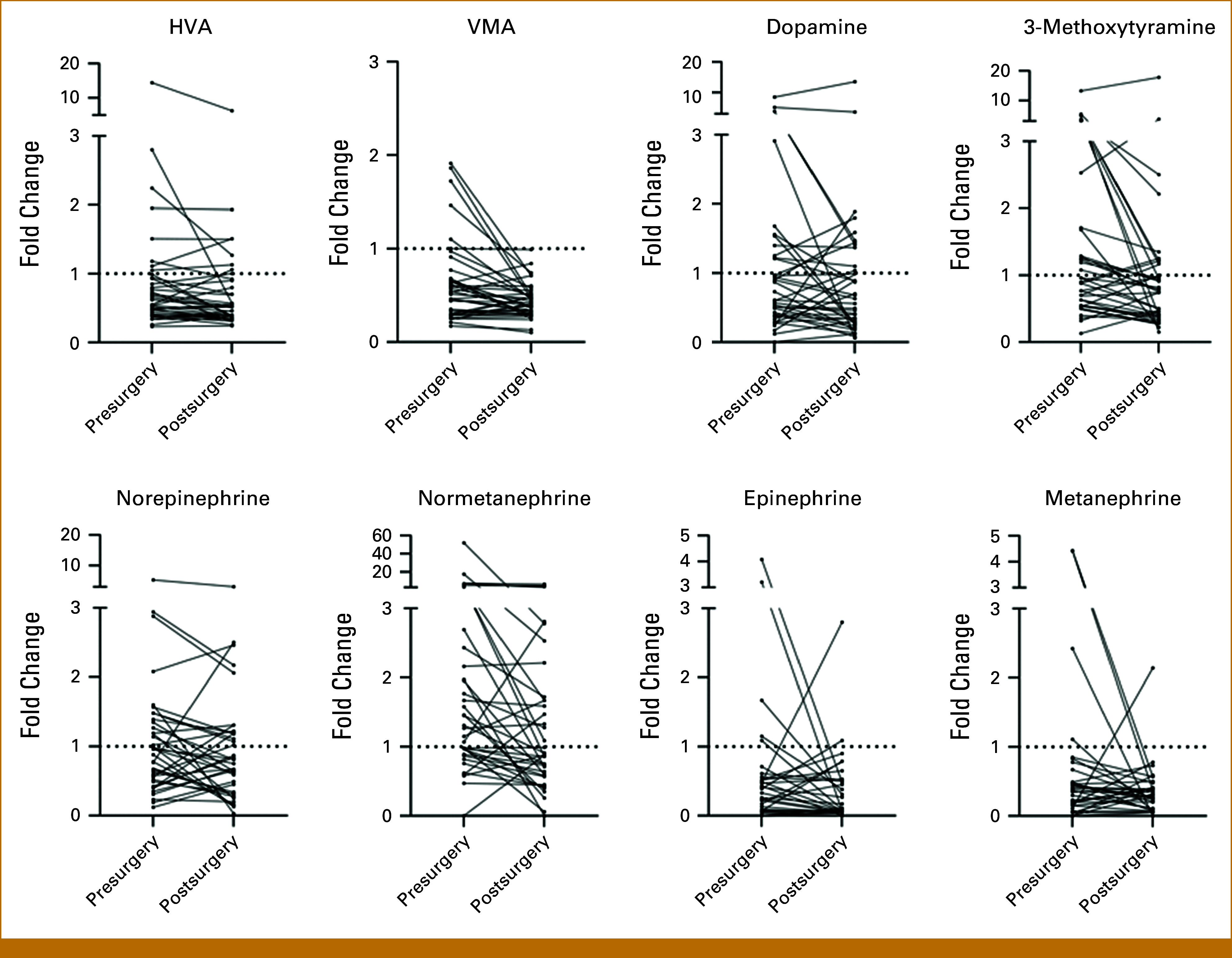
Differences in individual catecholamine levels before and after surgery. HVA, homovanillic acid; VMA, vanillylmandelic acid.

At the time of Dx, in 141 of 146 (97%) patients, at least one catecholamine metabolite was elevated. The percentage of patients with a positive catecholamine status declined during treatment, with 104 of 128 (81%) at PI, 69 of 93 (74%) at PC, 33 of 60 (55%) during maintenance therapy, and 15 of 71 (21%) at the ET (Appendix Fig A1). The fold changes of HVA, VMA, DA, 3MT, and NMN were highest (compared with other metabolites of the panel) at the time of Dx and showed a sharp decrease at PI, with levels further declining or stabilizing during the course of treatment (Fig 1). To investigate if the decline at PI versus Dx was affected by resection of the primary tumor (which coincided with this clinical time point), we analyzed 39 patients with paired catecholamine levels before and after resection of the primary tumor (Fig 2). Before surgery, 32 of 39 (82%) had a positive and seven (18%) had a negative catecholamine status. After surgery, 10 of 32 became negative, 22 of 32 remained positive, 3 of 7 became positive, and 4 of 7 remained negative (Fig 2A). Overall, 25 of 39 (64%) patients had a positive catecholamine status postsurgery. The metastatic lesions probably excrete catecholamines as postsurgery nuclear MIBG scans show significantly more metastatic lesions (defined by higher MIBG-SIOPEN scores) in the catecholamine-positive group (n = 25) compared with the catecholamine-negative group (n = 14; *P* = .04; Fig 2B). With regard to the individual metabolites, primary tumor resection resulted in a significant reduction in percentage of VMA-positive (*P* = .03) and NMN-positive patients (*P* = .02) and borderline significance for 3MT-positive patients (*P* = .05; Fig 2C; see Appendix Fig A2 for absolute differences in levels).

### Survival Analysis

**FIG 3.**
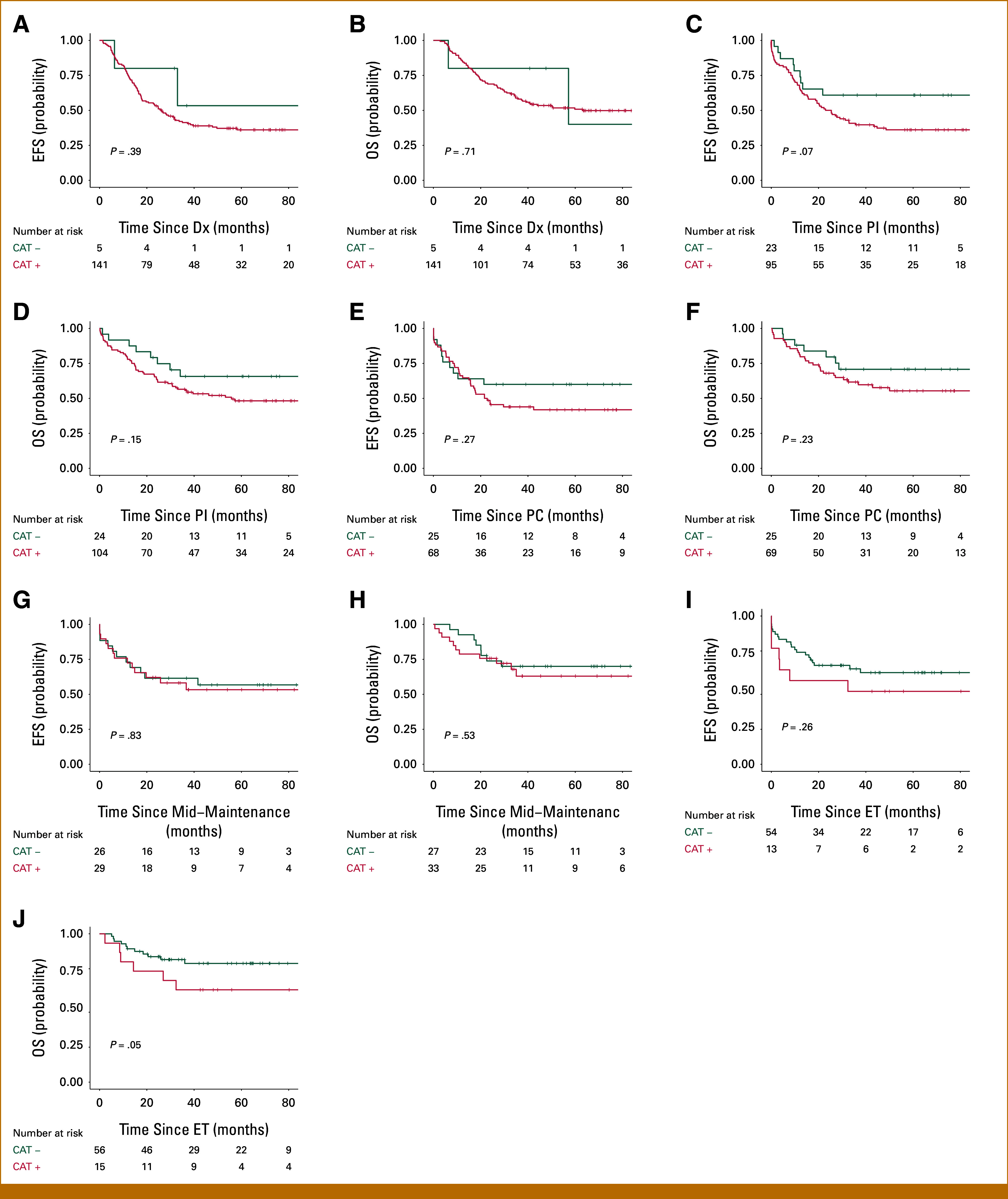
EFS and OS at Dx and during and after treatment. EFS and OS for patients with elevated and nonelevated catecholamine status from five clinical time points: (A and B) Dx, (C and D) postinduction, (E and F) postconsolidation, (G and H) mid-maintenance, and (I and J) at the end of treatment. The catecholamine status was considered elevated (CAT+) if any of the eight catecholamine metabolites exceeded the upper reference limit. CAT+, catecholamine status positive; CAT–, no elevation of any of the eight catecholamine metabolites; Dx, diagnosis; EFS, event-free survival; ET, end of treatment; OS, overall survival; PC, postconsolidation; PI, postinduction.

**FIG 4.**
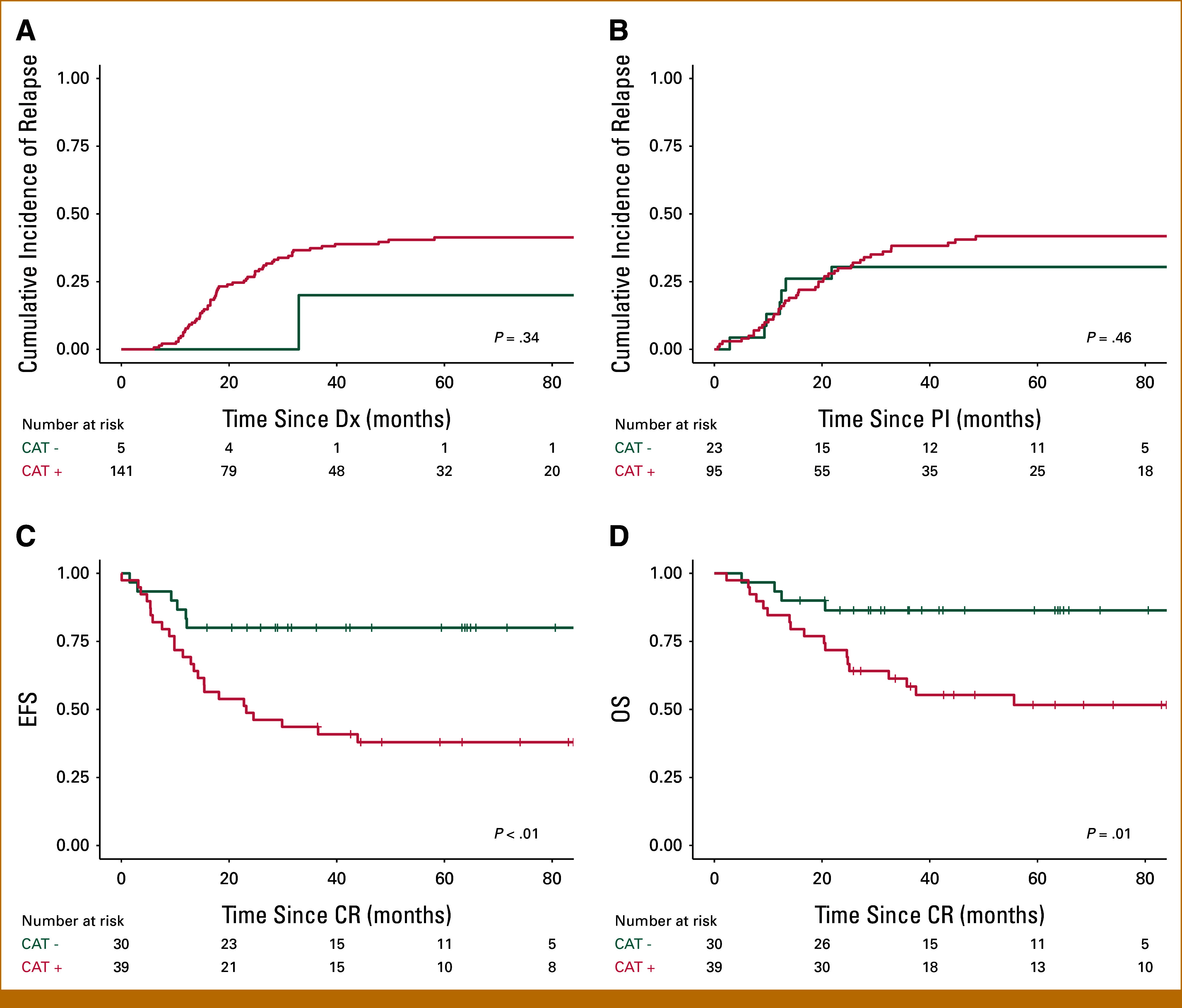
Cumulative incidence of relapse at Dx and PI and EFS and OS from CR. Cumulative incidence of relapse at (A) Dx, (B) at PI, (C) EFS from CR, and (D) OS from CR for patients with elevated (CAT+, red line) and nonelevated catecholamine status (CAT–, teal line). All events were relapses after CR was achieved. CAT+, catecholamine status positive; CAT–, no elevation of any of the eight catecholamine metabolites; CR, complete remission; Dx, diagnosis; EFS, event-free survival; OS, overall survival; PI, postinduction.

**FIG A3.**
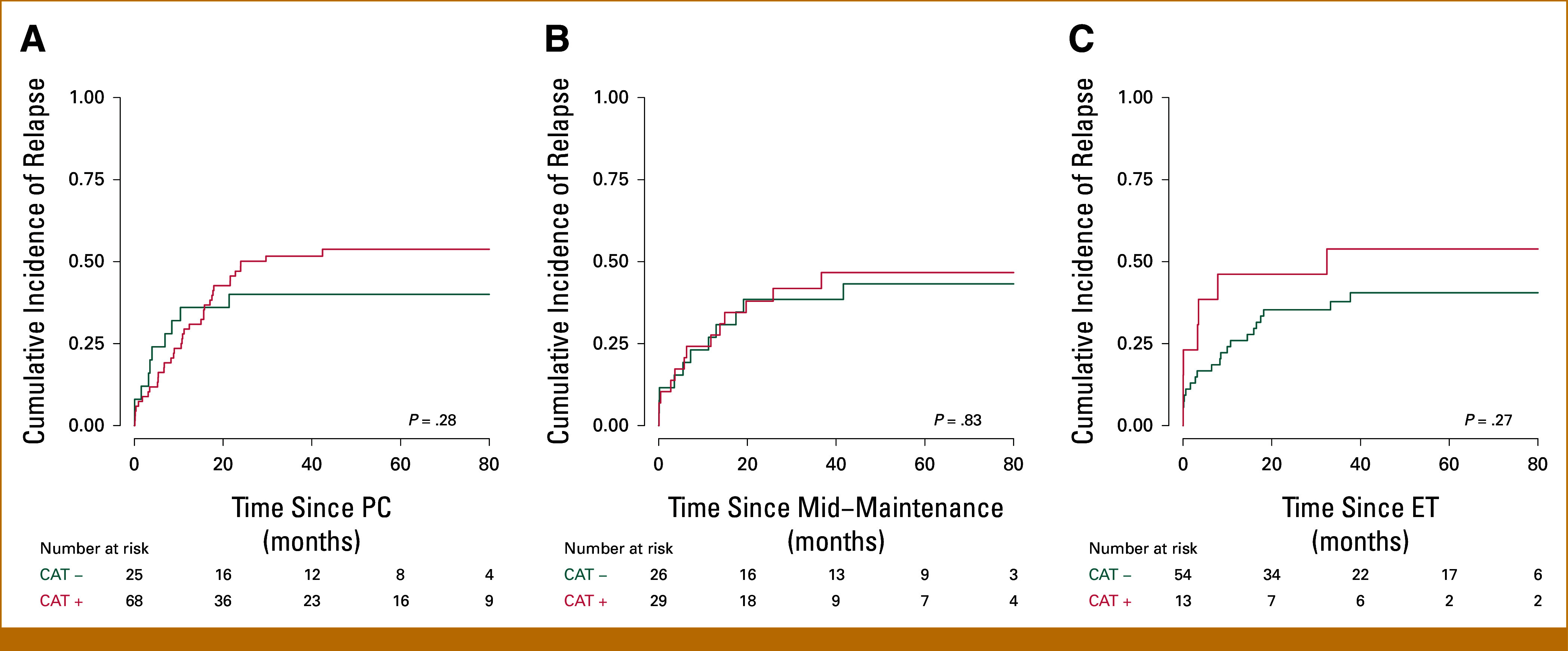
Cumulative incidence of relapse from PC, mid-maintenance, and the ET. CAT+, catecholamine status positive; CAT–, no elevation of any of the eight catecholamine metabolites; ET, end of treatment; PC, postconsolidation.

**FIG A4.**
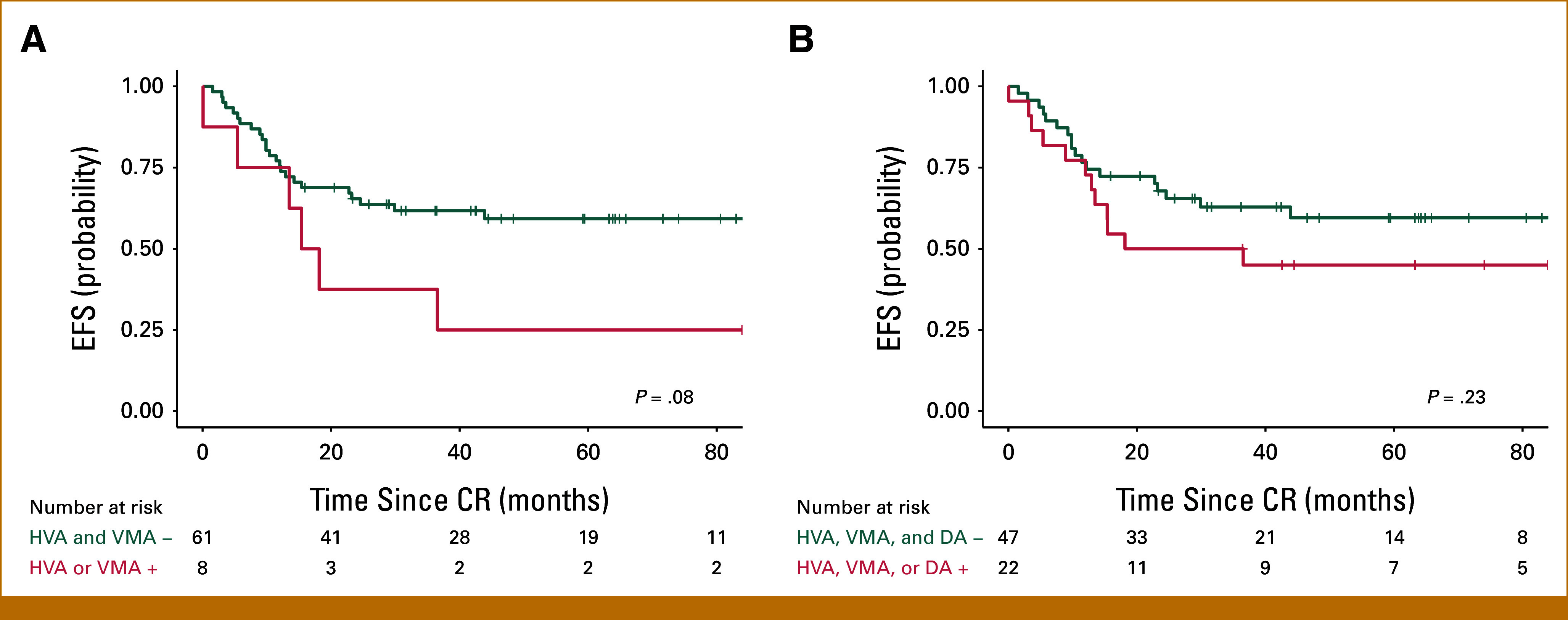
Relapse-free survival at CR for other catecholamine panels. CR, complete remission; DA, dopamine; EFS, event-free survival; HVA, homovanillic acid; VMA, vanillylmandelic acid.

EFS and OS for patients with a positive versus negative catecholamine status were assessed from different starting points: Dx, PI, PC, IT, and ET (Figs 3A-3J). Positive catecholamine status was not associated with poor survival outcomes at each of the time points compared with negative catecholamine status. However, catecholamine status at the ET was borderline significant for OS (*P* = .05; Fig 3J). Moreover, catecholamine status at any given time point was not a prognostic factor for cumulative incidence of relapse (Figs 4A and 4B; Appendix Fig A3). However, catecholamine status did contribute to determination of CR. Patients with a positive catecholamine status had poor EFS (all events were relapses) from CR (5-year EFS, 38% [95% CI, 0.25 to 0.57] for positive status *v* 80% [95% CI, 0.67 to 0.96] for negative status; *P* < .01; Fig 4C). The OS was also significantly affected, with a 5-year OS from CR of 52% (95% CI, 0.38 to 0.71) versus 86% (95% CI, 0.75 to 0.99) for positive and negative status, respectively (*P* = .01; Fig 4D). When comparing the performance of our panel with two commonly used catecholamine panels with fewer markers (the HVA/VMA panel and the HVA/VMA/DA panel), these were not predictive of EFS at CR (*P* = .08 and *P* = .23, respectively; Appendix Fig A4), indicating the added value of testing the extended catecholamine panel.

### Univariate and Multivariate Analyses

To study the association between catecholamine metabolite concentrations and survival outcomes, a univariate Cox proportional hazards regression model for each metabolite was estimated. The analysis was performed at Dx, PI, PC (Appendix Table A2), and at CR (Table 2). In the univariate analysis, at Dx, PI, and PC, several catecholamine metabolites were predictive of EFS and OS (Appendix Table A2). Most notably, higher levels of 3-methoxytramine were associated with EFS and OS at all three time points (hazard ratio [HR] for OS, 2.2 [95% CI, 1.4 to 3.4], 3.1 [1.6 to 5.9], and 3.2 [1.5 to 6.9] at Dx, PI, and PC, respectively). At CR, higher levels of DA, 3MT, NE, and NMN were significantly associated with both poor EFS and OS (Table 2). A positive catecholamine status at CR was a significant independent prognostic value for EFS (HR, 4.4 [95% CI, 1.8 to 10.7]) and OS (HR, 4.0 [95% CI, 1.4 to 11.7]; Table 2), with *MYCN* amplification and age included in both models.

**TABLE 2.** Cox Proportional Hazards Regression Model for Relapse-Free Survival and OS Analyzed From CR

Univariate Model (metabolites)	Event-Free Survival, HR (95% CI)	OS, HR (95% CI)
Homovanillic acid	3.3 (1.2 to 9.5)*	2.5 (0.9 to 7.1)
Vanillylmandelic acid	3.1 (0.9 to 10.5)	2.5 (0.6 to 11.1)
Dopamine	3.5 (1.0 to 12.2)*	5.9 (1.2 to 28.3)*
3-Methoxytyramine	4.8 (1.3 to 18.0)*	7.5 (2.0 to 28.6)*
Norepinephrine	4.7 (2.0 to 11.1)*	5.8 (2.2 to 15.4)*
Normetanephrine	3.0 (1.4 to 6.7)*	3.2 (1.2 to 8.1)*
Epinephrine	1.6 (0.7 to 3.6)	1.3 (0.5 to 3.6)
Metanephrine	1.6 (0.6 to 4.7)	1.4 (0.4 to 5.1)
Multivariate model (prognostic factor)		
Age >18 months *v* <18 months	3.1 (1.0 to 10.1)*	2.9 (0.7 to 12.6)
*MYCN* amplified *v* nonamplified	1.3 (0.6 to 2.6)	1.4 (0.6 to 3.2)
Catecholamine status at CR positive *v* negative	4.4 (1.8 to 10.7)*	4.0 (1.4 to 11.7)*

NOTE. Continuous catecholamine values were used for the univariate Cox proportional hazards regression model. All events were relapse events after CR was reached.

Abbreviations: CR, complete remission; HR, hazard ratio; OS, overall survival.

*A significant test with *P* < .05.

## DISCUSSION

In this study, we analyzed a panel of eight urinary catecholamine metabolites over the course of treatment in patients with high-risk neuroblastoma. With ongoing treatment, catecholamine levels decreased over time, with a notable decrease in catecholamine concentrations after surgery. Most importantly, our study showed that catecholamine status contributed to the establishment of CR and independently predicted relapse and survival outcomes. At Dx, and at standard evaluations and clinical decision making moments during treatment, catecholamine status was not associated with survival outcomes. However, individual metabolite concentrations, most notably 3MT, were predictive of poor EFS and OS at Dx, PI, and PC.

The therapy-induced overall decrease in catecholamine metabolite levels during treatment in our cohort is consistent with earlier findings.^17,23,24^ The decrease in catecholamine levels after six courses of chemotherapy in our study is in line with the findings of a previous study after four initial courses of chemotherapy without surgery, in which 37% of patients remained positive for HVA or VMA, compared with 91% at Dx.^17^ In our study, the decline in metabolite levels can be explained by both the response of metastatic lesions to systemic therapies and the surgical removal of the primary tumor. We showed that in 64% of patients who were catecholamine-positive after resection, significantly more MIBG-positive neuroblastoma skeletal lesions were detected. However, we cannot exclude any postoperative residue of the primary tumor contributing to catecholamine excretion.^25,26^ Furthermore, our panel consists of eight markers, which was shown to increase the diagnostic sensitivity by 11% compared with the two-marker panel of HVA and VMA.^13^ This increased sensitivity might also explain the 64% panel positivity after induction therapy and surgery.

Previous studies investigated the relationship between catecholamine metabolites and survival in patients with neuroblastoma. Persistent elevations of VMA, HVA, and total MNs were present in most neuroblastoma nonsurvivors, whereas survivors without evidence of disease mostly had normalized catecholamine levels.^24,27,28^ Our study provides information about the effect of catecholamines on time-to-event analysis, whereas the previous studies only reported descriptive data.^24,27,28^ Hero et al^17^ conducted survival studies where urinary HVA and VMA, measured after four courses of chemotherapy, were not associated with EFS. Of note, they furthermore showed that elevated plasma HVA and VMA did show a negative association with EFS.^17^ Similar to their findings, our extended catecholamine panel was not significantly associated with outcomes at the end of induction. However, catecholamine status at the ET was borderline significant for OS. Given the uncertainty in administering additional treatment at the end of the maintenance phase, these results warrant validation studies conducted in larger cohorts. If the results can be validated, patients with persistently elevated metabolites might benefit from further treatment, such as temozolomide, or vaccines (ClinicalTrials.gov identifier: NCT00911560) that are being investigated in clinical trials.^29,30^

Our study demonstrated the added value of measuring catecholamine metabolites for predicting patient outcomes at the time of CR. Lam et al^23^ reported overall normalized levels of DA, HVA, VMA, and 3MT in patients who completed treatment and showed no radiologic evidence of tumor activity (ie, CR). By contrast, we observed elevated catecholamine levels in 55% of patients at CR. A possible explanation for differences with the results from the study by Lam et al^23^ may be due to the number of catecholamines included in the analysis as we measured eight metabolites which might have increased the sensitivity. Furthermore, we determined whether patients were in CR on the basis of the INRC during treatment, whereas Lam et al^23^ analyzed patients after treatment was finished. Several studies compared urinary catecholamines with other modalities, including bone marrow studies and MIBG scans, and showed that HVA and VMA have low sensitivity as markers for monitoring disease activity in patients with neuroblastoma.^17,31-35^ This resulted in the removal of HVA and VMA from the INRG response criteria in 2017.^11^ Previous studies showed the added value of measuring an extended panel of catecholamine markers, compared with HVA and VMA for diagnostic and prognostic purposes.^13,15,16,36^ In this study, HVA and VMA were not associated with EFS at CR, highlighting the importance of including additional catecholamines. However, we observed that some markers, such as E and MN contribute to a small extent, suggesting that the panel could potentially be reduced to a more limited set of markers. Furthermore, the individual metabolites were strong prognostic markers at several time points during therapy, especially 3MT. Previously, 3MT was established as an important diagnostic, prognostic, and response monitoring biomarker and we showed a clear link between elevated 3MT levels and MYC activity.^15^ Future studies are warranted to assess the role of the panel of eight catecholamine metabolites compared with, or together with, other modalities. Within SIOPEN, the catecholamine working group aims to harmonize catecholamine measurements while validating their metabolites as diagnostic,^14^ prognostic, and response markers, alongside investigating their association with biologic processes.^15^ Most SIOPEN centers currently use certified assays for catecholamine measurements, which facilitates broad implementation in clinical care.

This retrospective study was limited by the relatively small sample cohort and retrospective design. In addition, at some clinical time points, up to 50% of urine samples were unavailable. The higher number of missing urine samples may be due to urinary catecholamine metabolites not being included in the current INRC criteria, making their collection less prioritized. The missing urine samples were random. We observed that the time points at which patients achieved CR during the study period were similar between those included in the analysis and those with missing data. Of note, we found similar rates of CR (at PI and before immunotherapy) compared with other studies.^3,37-39^

In conclusion, urinary catecholamine metabolites are easily measurable noninvasive biomarkers during the course of neuroblastoma treatment and the eight-marker panel is associated with poor survival in high-risk patients. Furthermore, our study showed that catecholamine detection is a useful addition to the current criteria for complete response as catecholamine metabolites have additional prognostic value. Future studies should evaluate the role of the eight-marker catecholamine panel in disease monitoring and establishing CR in high-risk neuroblastoma, especially compared with other current disease monitoring modalities.

## Data Availability

A data sharing statement provided by the authors is available with this article at DOI https://doi.org/10.1200/PO-24-00491.

## APPENDIX

**TABLE A1.** CR Status at Each Clinical Time Point

Catecholamine Metabolites	Total No. of Patients	Patients Included for Analysis
Postinduction	53	29
Postconsolidation	33	22
Mid-maintenance	14	7
End of treatment	20	11
Total	120	69

NOTE. Number of patients who reached CR at each clinical time point during and after treatment. Patients were included for analysis if urinary catecholamine data were available.

Abbreviation: CR, complete remission.

**TABLE A2.** Univariate Cox Hazard Regression Model for EFS and OS at Dx, PI, and PC

Catecholamine Metabolites	EFS, HR (95% CI)			OS, HR (95% CI)		
	Dx	PI	PC	Dx	PI	PC
HVA	1.4 (0.9 to 2.2)	2.9 (1.5 to 5.8)*	2.1 (0.8 to 5.1)	1.8 (1.0 to 3.0)	3.4 (1.8 to 6.5)*	1.9 (0.6 to 5.5)
VMA	0.9 (0.7 to 1.4)	1.5 (0.8 to 2.7)	2.0 (0.6 to 7.1)	1.0 (0.7 to 1.5)	2.1 (1.1 to 3.9)*	1.5 (0.3 to 6.8)
DA	1.4 (0.9 to 1.9)	1.5 (0.8 to 2.7)	2.4 (0.9 to 6.2)	1.4 (0.9 to 2.1)	1.9 (1.0 to 3.6)*	2.6 (0.9 to 7.6)
3MT	1.8 (1.2 to 2.6)*	2.2 (1.2 to 4.0)*	2.7 (1.3 to 5.5)*	2.2 (1.4 to 3.4)*	3.1 (1.6 to 5.9)*	3.2 (1.5 to 6.9)*
NE	1.8 (1.2 to 2.8)*	1.7 (0.9 to 3.4)	2.4 (1.0 to 5.7)*	2.0 (1.3 to 3.1)*	2.5 (1.2 to 5.3)*	1.9 (0.7 to 5.2)
NMN	1.2 (0.9 to 1.6)	1.8 (1.1 to 2.9)*	2.3 (1.0 to 5.0)*	1.3 (1.0 to 1.8)*	2.3 (1.4 to 3.7)*	2.1 (0.8 to 5.3)
E	1.2 (0.8 to 1.8)	0.7 (0.4 to 1.2)	0.7 (0.4 to 1.4)	1.2 (0.7 to 1.9)	0.7 (0.4 to 1.4)	0.8 (0.4 to 1.7)
MN	0.9 (0.5 to 1.5)	0.8 (0.4 to 1.5)	0.9 (0.5 to 1.9)	0.9 (0.5 to 1.5)	1.1 (0.6 to 2.2)	1.0 (0.5 to 2.4)

NOTE. HR and 95% CIs are shown. Continuous catecholamine values were used for this model. This analysis was not performed for mid-maintenance and end of treatment because of the small number of events.

Abbreviations: 3MT, 3-methoxytyramine; DA, dopamine; Dx, diagnosis; E, epinephrine; EFS, event-free survival; HR, hazard ratio; HVA, homovanillic acid; MN, metanephrine; NE, norepinephrine; NMN, normetanephrine; OS, overall survival; PI, postinduction; PC, postconsolidation; VMA, vanillylmandelic acid.

*A significant test with *P* < .05.
